# Dry Panels Supporting External Quality Assessment Programs for Next Generation Sequencing-Based HIV Drug Resistance Testing

**DOI:** 10.3390/v12060666

**Published:** 2020-06-20

**Authors:** Marc Noguera-Julian, Emma R. Lee, Robert W. Shafer, Rami Kantor, Hezhao Ji

**Affiliations:** 1 IrsiCaixa AIDS Research Institute, Hospital Germans Trias i Pujol, s/n, Catalonia, 08196 Badalona, Spain; 2 Chair in AIDS and Related Illnesses, Centre for Health and Social Care Research (CESS), Faculty of Medicine, University of Vic, Central University of Catalonia, Can Baumann. Ctra. de Roda, 70, 08500 Vic, Spain; 3 National HIV and Retrovirology Laboratories, National Microbiology Laboratory at JC Wilt Infectious Diseases Research Centre, Public Health Agency of Canada, Winnipeg, MB R3E 3R2, Canada; emmar.lee@canada.ca (E.R.L.); hezhao.ji@canada.ca (H.J.); 4 Stanford University Medical School, Stanford, CA 94305, USA; rshafer@stanford.edu; 5 Division of Infectious Diseases, Brown University Alpert Medical School, Providence, RI 02903, USA; rkantor@brown.edu; 6 Department of Medical Microbiology and Infectious Diseases, Rady Faculty of Health Sciences, University of Manitoba, Winnipeg, MB R3E 0J9, Canada

**Keywords:** HIV, drug resistance testing, next generation sequencing, external quality assessment, dry panel

## Abstract

External quality assessment (EQA) is a keystone element in the validation and implementation of next generation sequencing (NGS)-based HIV drug resistance testing (DRT). Software validation and evaluation is a critical element in NGS EQA programs. While the development, sharing, and adoption of wet lab protocols is coupled with the increasing access to NGS technology worldwide, rendering it easy to produce NGS data for HIV-DRT, bioinformatic data analysis remains a bottleneck for most of the diagnostic laboratories. Several computational tools have been made available, via free or commercial sources, to automate the conversion of raw NGS data into an actionable clinical report. Although different software platforms yield equivalent results when identical raw NGS datasets are analyzed for variations at higher abundance, discrepancies arise when variations at lower frequencies are considered. This implies that validation and performance assessment of the bioinformatics tools applied in NGS HIV-DRT is critical, and the origins of the observed discrepancies should be determined. Well-characterized reference NGS datasets with ground truth on the genotype composition at all examined loci and the exact frequencies of HIV variations they may harbor, so-called dry panels, would be essential in such cases. The strategic design and construction of such panels are challenging but imperative tasks in support of EQA programs for NGS-based HIV-DRT and the validation of relevant bioinformatics tools. Here, we present criteria that can guide the design of such dry panels, which were discussed in the Second International Winnipeg Symposium themed for EQA strategies for NGS HIVDR assays.

## 1. Introduction

Drug resistance testing (DRT) methods for HIV-1 are continually evolving as exemplified by NGS technology, which is gradually replacing Sanger chemistry in clinical diagnostics [1,2]. NGS has the capability to generate high-quality genotypic data at a low cost when a large volume of samples is available, with a relatively shorter turn-around time and higher sensitivity compared to traditional Sanger methods. NGS technology is versatile and can deal with different specimen types and HIV-1 subtypes, producing an unprecedented high volume of data. It is able to detect low abundance viral variants, such as minority resistant variants (MRV), that are not readily detectable by Sanger methods, with potential added value for clinical management [3,4,5,6]. However, the analysis of the large volumes of raw NGS data generated in such tests requires complex and reproducible bioinformatic tools to provide robust and clinically actionable results. Therefore, the capacity of a laboratory in the effective management of NGS data constitutes an essential component in external quality assessment (EQA) of the laboratory’s competence in performing NGS-based HIV-DRT.

Reference materials or proficiency test (PT) panels have long been used in the validation of experimental procedures and EQA applications for laboratories performing Sanger-based HIV-DRT [7,8,9]. While validated experimental methods are essential to EQA, every execution of a wet lab protocol may result in variations in the derived output dataset, which may lead to discrepancies in the downstream analysis. Because of the high sensitivity and quantitative nature of NGS-based HIV-DRT, such differences are of more significance than in Sanger-based technologies. Converting large volume raw NGS data, of different quality, into end-user interpretable HIV-DRT reports is, thus, a challenging task. Various noise and errors resulting from the laboratory procedures may significantly hamper the design, optimization, and performance of software tools, and eventually, the competence of a laboratory in conducting NGS DRT. Due to the limited capacity of a wet panel in assessing the particular lab ability for effective NGS HIV-DRT data management, well-characterized reference in silico NGS datasets with known ground truth on the genotype composition at all examined loci and the exact frequencies of HIV variations they may harbor, so-called dry panels, are urgently required but yet to be developed.

Software pipelines that support NGS HIVDR assays are required to perform a series of data analytical steps to covert raw NGS data into an HIV drug resistance report. While some variations exist, these pipelines share many essential procedures, including filtering of short and poor quality reads, reference alignment, variant calling, drug resistance mutation (DRM) identification, query against selected clinically validated algorithm(s), and a final data reporting step [10,11,12]. Many bioinformatic pipelines are currently available with different degrees of complexity in their use [13,14,15,16,17]. While a simple-to-use interface is preferred, especially for diagnostic use, methods for the specific validation of the different algorithmic approaches and the corresponding implementation and execution by the final user are needed. That is, the accuracy of the algorithm needs to be verified so that the final output can be guaranteed to an end-user, as high-quality results. The validation of the existing algorithms largely relies on the analysis of plasmid derived NGS, which is not representative of the complexity of data from real clinical specimens. Besides its foreseeable EQA application, well-characterized dry panels consisting of data from different samples, of different quality, and representative of the major NGS platforms, would certainly benefit the development and refinement of software tools and serve the technical training needs for conducting such analysis properly.

## 2. Goals of NGS Dry Panels for HIV-DRT

The primary applications of a dry panel in genotypic assays include: (1) facilitating the development, refinement, and validation of effective data processing software or bioinformatics tools; (2) enabling technical training and troubleshooting on issues that arise from non-lab procedures; and lastly but most importantly; (3) allowing EQA assessment of the lab competence in relevant data management while involving no real sample processing. Therefore, well-designed dry panels for a genotypic assay should include reference sequences representing raw sequencing outputs containing all major variations, possible artificial biases, and uncommon sequencing errors one may encounter while processing genuine NGS HIV-DRT data.

The ground truth or reference values for the subjects of interest (i.e., genotypes, mutations, and their expected frequencies) should be pre-determined, against which downstream comparative analyses could be conducted. The ground truth represents the reference values against which the specificity, sensitivity, and additional accuracy and precision quantitative measures could be determined. These can include a reference resistance genotype, a genetic sequence, the presence/absence of specific mutations, or additional information regarding the quality grade of the data.

In addition to standard PT specimen panels, capacity building and EQA programs for Sanger-based HIV-DRT have previously benefited from the use of data dry panels [18,19,20,21,22,23], by sharing specific electropherogram and sequence data with linked ground truth. For that, dry panels for SS HIV-DRT include not only regular SS files containing specific HIV DRMs, but also datasets of low quality or erroneous data that approximate typical quality problems one may encounter in the use of this technology. Within the contexts of EQA programs, these panels help to specifically assess data analysis capabilities of testing laboratories to reliably detect DRMs by interpreting the same sequencing data, since raw SS data files are provided instead of biological specimens and, thus, comparisons are not affected by the intrinsic variability of wet lab techniques and protocols.

Similar to SS, to-be-developed dry panels for NGS DRT are expected to satisfy all alike needs with additional capacity in the quantitative assessment of NGS output for HIV DRM detection. In addition, NGS dry panels should also represent NGS data variability, with a broad scope of potential errors and artifacts that may arise from the manipulation of samples in NGS experimental protocols. The main goals of an HIV-DRT NGS dry panel are to: 1) test the ability of a bioinformatic tool/pipeline to reliably predict a resistance profile; 2) accurately detect HIV DRMs in both qualitative and quantitative manners; and 3) comply with a standard of quality while being representative of the different NGS technologies and the application context, including error profiles of sequencing platforms, HIV diversity and variability, and experimental design.

**Figure 1:**
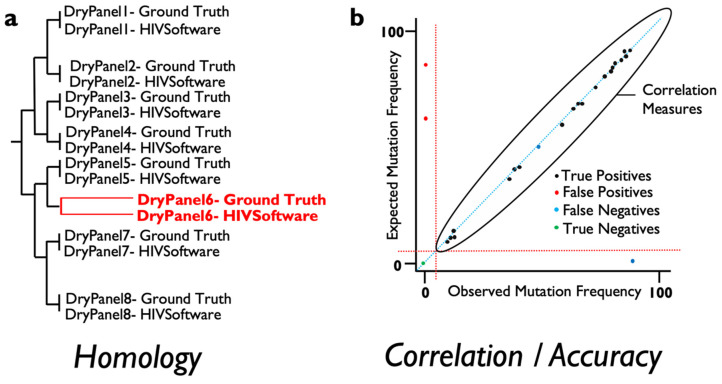
Types of proposed accuracy measures to be evaluated for next generation sequencing (NGS)-based HIV drug resistance testing (DRT) dry panels. (**a**) Using NGS-derived consensus sequences, a phylogenetic tree can be calculated with ground truth and software results in order to assess phylogenetic identity; (**b**) Using mutation frequencies as detected in NGS data, mutations can be classified as detected/not-detected at specified thresholds and derived accuracy measures can be used to validate software tools. In addition, a direct correlation using ground truth and obtained mutation frequencies can be calculated for each sample or samples group.

For this, several measures of agreement between ground truth and the results derived from varied software can be obtained and automatically implemented to score the validity of results when NGS data are analyzed. At the consensus (Sanger-like sequence) level, a single sequence representing the whole NGS-based viral population, the number of discrepancies or nucleotide mismatches between the ground truth sequence, and the obtained sequence should ideally be 0 in terms of similarity across the genome region examined. In addition, a measure of similarity can be measured for every pair of ground truth result data to detect those sequence datasets where the analysis has failed to provide an equal representation of the consensus sequence for the viral population. These measures include phylogenetic distances and tree building that include within-codon mixtures or the calculation of the proportion of differences (Figure 1a). On the other hand, in order to assess whether the frequency of the mutations detected by a specific software is accurate, standard correlation, specificity and sensitivity analysis can be performed to ensure that results are above a pre-defined threshold analysis (Figure 1b). Linking failures to particular features in the dry panel dataset can help determine which steps need revision in the evaluated software or testing laboratory.

The First Winnipeg Consensus proposed prototypic guidelines specific to NGS data analysis for HIV-DRT, which may serve as a scaffold when designing dry panels for such assays [10]. It specified the essential steps and criteria for relevant data management, including: (1) quality controls, incorporating low quality and contamination detection; (2) sequence read alignment strategies for HIV-1 protease, reverse transcriptase, and integrase proteins; (3) variant calling and quality control based on these alignments; (4) variant interpretation and reporting; and (5) general data management. Accordingly, dry panel datasets need to be designed to challenge the analysis process in detecting both specific features within the sequence data and how these data are analyzed that can result in artifactual results when real data are used for diagnostics. A dry panel should satisfy the needs for assessing and validating all steps in an analysis pipeline including identifying and filtering out low quality data and ruling out false drug resistance mutations caused by signature hypermutation, erroneous base-calling, or improper alignments (Table 1).

## 3. Challenges for NGS Dry Panel Development

### 3.1. NGS Accuracy Assessment

The sensitivity and specificity assessment of NGS HIV-DRT pose different challenges, compared to SS HIV-DRT, because of the quantitative nature and higher sensitivity of such assays [24]. NGS HIV-DRT is privileged to have the capacity to detect MRVs in the viral population. SS can arguably detect viral variants down to the ~15% of abundance and the dry panels used for SS HIV-DRT are qualitative or semi-quantitative by nature. They are able to detect the presence/absence of a mutation but not quantify its frequency in the viral population. Conversely, NGS HIV-DRT detects DRMs at a much lower prevalence and quantifies their frequency within a viral population. The technical threshold for DRM detection depends on the experimental procedures used to obtain the NGS dataset and is usually defined based on positive DNA controls. This threshold often lies well below reporting thresholds for clinical use [4,25]. Thus, EQA for NGS is quantitative and needs to specifically assess the ability of software tools to determine the frequency of a particular mutant, without the variability of the experimental protocol that is inherently linked to the generation of such data. For that, the definition of what is the ground truth for a specific dataset needs to be established based on the accuracy and precision of a particular data analysis tool or the performance of an EQA-evaluated laboratory.

### 3.2. Sequencing Technologies and Experimental Approaches

Currently, Illumina and Ion Torrent are the dominant NGS technologies used for HIV-DRT [26,27]. Each of them has its specific error profile and characteristics that need to be considered during analysis. In other words, a bioinformatic tool that provides robust results on a dataset from one specific NGS technology may fail to provide reliable output for other technologies. Therefore, all main NGS platforms need to be represented in NGS dry panels if they are to be used in a multi-platform context to ensure the adequate dataset option of choice for specific laboratories in the context of EQA programs.

In addition, several experimental strategies exist for NGS sequencing of the HIV genome. For instance, genome sequencing- or amplicon sequencing-based experimental designs may show different features in the data that result in different biases in terms of sequence duplication, recombination, or template sampling. It is also common to aim for different depths of coverage or use diverse sequencing lengths in order to balance laboratory costs. All such factors should be taken into consideration while constructing the perspective dry panels for NGS HIV-DRT. Unique Molecular Identifiers have also been proposed as a way to overcome the bias introduced at the DNA amplification steps and correct for sequencing error, provided that specific data analysis steps are used [28,29]. Thus, the ability to test any software to adapt to and address challenges specific to particular experimental designs is also needed, the assessment of which requires exemplar reference datasets that approximate those from different experimental approaches.

### 3.3. Data Availability and Exchange Formats

The data format of dry panel files needs to be readily usable by all different software platforms. FASTQ and SAM/BAM are used by most common sequencing platforms and are suitable for data exchange. On the other hand, to be able to exchange and evaluate results in a timely and reproducible manner, the amino acid variant format (AAVF) was defined by the First Winnipeg Consensus [30]. This format was designed to keep the codon-level results and all associated metadata. Parsers and format compliance checking tools were also provided as open-source software. The Winnipeg Consensus advised that newly developed software tools aimed for HIV NGS-based genotyping validation should produce results in this format to facilitate quality assessment processes. Finally, FASTQ files will need to be provided as downloadable files shared through public data servers or specific repositories such as HIVdb. This would be accompanied by the creation of evaluation methods using the tools as mentioned above on AAVF outputs from the client labs. Notably, steady access to internet is required for both the participating labs, for reference datasets downloading and analysis data submission, and the EQA administrator, who require web-based servers for handling large data files during the program operation.

## 4. Dry Panel Data Types

### 4.1. Real Data Dry Panels

Real data are the primary real-world source of data for HIV-DRT bioinformatic tools. Importantly, real data harbors actual errors and artifacts derived from experimental protocols. Real data can be obtained from HIV RNA extracted from patient samples, HIV culture supernatants, or HIV DNA using commercially available HIV plasmids, commonly used in most diagnostic labs as positive controls. Each of these genetic material sources shows specific features in terms of quality control. For instance, plasmid-derived NGS data do not show errors linked to the reverse transcription step needed to process RNA samples.

The use of plasmids or RNA from culture genetic material provides a way to create artificial viral mixtures with controlled MRV abundance of interests. Still, the quantification of such mixtures shows intrinsic error and variability. Thus, its link to the readouts from different data analysis tools on the same NGS dataset and its usability as ground truth can’t be derived solely from those measured concentrations.

Similarly, one of the main disadvantages of using real datasets for dry panel design is that the ground truth of the exact frequencies of the DRMs they harbor cannot be reliably determined. As an approximation to ground truth, the majority vote consensus of results obtained from several well-established tools could be used to establish the ground truth which could then be used to measure the accuracy of additional tools as they become available [31]. This is especially important when trying to assess the presence of MRV (those between 1% and 15%) where most discrepancies among the available software are found.

Once a methodology has been established to define ground truth for real data by using statistical agreement, datasets obtained from clinical specimens, viral culture, or plasmid samples can be obtained and analyzed through generic or HIV-DRT specific repositories such as NCBI/SRA and EMBL/ENA or HIVdb-NGS [12], respectively, to contribute to dry panel construction. These data are suitable to test bulk batches of sample datasets with evident errors or quality problems and to test new tools for accuracy in describing highly prevalent viral mutations.

### 4.2. Synthetic and in Silico Dry Panels

Aside from real-world data, the generation of synthetic data has been widely used to test software in all biomedical fields using NGS technologies [32,33,34]. A myriad of software tools is available that are capable of mimicking the sequence-specific error (SSE) profiles of the different NGS platforms. These tools can also model different experimental designs or organism-linked mutation generation models to represent mixtures. In addition, they are able to include mutation and recombination events, based on statistical models alone or a combination of statistical models with parametrization obtained from real data. Therefore, quality, mutation frequencies, mixtures and contamination can be modelled to create input files for synthetic dry panels. Nevertheless, these data are still a result of a model and may fail to account for the artificial errors or amplification biases introduced during the wet lab procedures, especially the PCR steps, which are drastically different from SSEs and should be properly managed by the data analytics tools. This represents one of the main weaknesses of such a strategy for dry panels since a software tool that performs well on model-derived data may show a decreased performance on real data.

A forged dry panel can be described as a carefully crafted combination of real data sequence reads in order to create real datasets with a known ground truth supporting them. The main idea behind a forged dry panel is the careful annotation of NGS sequence data at the read level, by linking each read to the presence/absence of mutations, technology, experimental design, quality score, HIV-1 genome region, and other artifacts that reads harbor. By adding up all these read-level annotations, a composite read data library can be built with thousands or millions of reads that can be combined into forged sample datasets by selecting the reads with the properties expected to be tested in the dry panel. In this way, the real error profile is kept and the specific features to be tested can be combined in a controlled manner.

## 5. Design of Dry Panels

To date, most of the available software tools for NGS-based HIV-DRT have been validated through internally generated datasets. Few studies have reported validation using different data analysis methods and often comparing different sequencing platforms [35,36]. While studies regarding the comparison of data analysis procedures for NGS HIV-DRT are scarce, recent reports on the cross-validation of various data analysis pipelines on the same real datasets show an excellent agreement between different pipelines when a modest sensitivity threshold (>2%) is required [31,37]. Lee et al. used datasets obtained from several different laboratories on the same set of PT specimens and cross-analyzed them using five available analysis platforms. Ground truth for these samples was defined on a majority vote basis, defining a mutation present if 4 out of 5 analysis platforms detected it. Similarly, Jair et al. validated publicly available pipelines with similar results on real HIV NGS data. In addition, abundant and diverse HIV NGS real data are available at public repositories such as NCBI/SRA or EMBL-EBI/ENA, shared by HIV studies involving software development or epidemiological characterization [38]. These include most of the available NGS platforms and datasets representative of HIV diversity and can be easily accessed and used through appropriate mechanisms for dry panel design purposes. However, the diversity of experimental protocols may be linked to specific features data analysis pipelines that are suited to protocol-derived features but not to others. Thus, the testing of these may require additional datasets to correctly validate these pipelines.

A comprehensive dry panel should be able to test all of the required quality criteria in a minimal dataset. The First Winnipeg Consensus outlined the essential data analysis steps for NGS HIV-DRT [10]. Accordingly, the prospective dry panel may be constructed to address the need for testing the capacity of the bioinformatics tools in performing these designated tasks (Table 1).

For sequence quality control, short or low-quality sequence reads can be included that harbor a specific signal mutation that should not pass acceptable quality filters. Similarly, highly contaminated or cross-contaminated samples can be included in the dry panel dataset to ascertain if the analysis software can correctly detect and/or describe sample contamination and its source.

The sequence alignment strategy is a central point in NGS-based HIV-DRT and also needs to be considered for dry panel design. Many alignment algorithms and derived software tools exist that can be used for HIV-1-DRT. The performance of these alignment tools may vary depending on the error profiles of varied NGS platforms and on the genetic diversity of the examined genomic regions [39]. Aside from the strategy of choice, alignment should be performed using the HXB2 HIV-1 reference [40] or a sample specific consensus *pol* gene sequence, albeit this may not be a requirement for some analysis strategies. HXB2 is suggested as an aligner reference based on considerations including: (1) HIV-1 *pol* gene is highly conservative among all HIV subtypes, for which the choice of reference has minimal impact on subsequent alignment; (2) HXB2 is a common coordinate system for HIV-1 DRM calling and reporting; (3) the use of a “unified” aligner reference facilitates inter-lab or inter-pipeline comparisons during EQA. Insertions and deletions with respect to a reference are especially challenging to detect and manage, which can benefit from codon-aware alignment strategies and improved alignment quality control [41,42]. Both in-frame and out-of-frame insertions and deletions should be included in the dry panel datasets for assessing the pipeline capacity in properly managing such challenging sequence variations.

For the variant calling quality control step, the Winnipeg Consensus recommended using codon-level variant calls and reporting, advising against the use of consensus sequence to represent NGS-based drug resistance data. This is because mixed bases present at two or more loci within a codon may result in artifactual or assumptive mutations if only data at consensus level are interpreted. Inclusion of such within-codon mixes is therefore advised to check whether the analysis software is treating variants at the codon-level or nucleotide-level. Additional controls for variant calling include minimum count number of specific mutations and minimum coverage to call variations at a specified threshold since sensitivity is dependent on the depth of coverage.

In order to perform an accuracy/precision assessment of the results, the most important measure is the agreement between the obtained drug resistance profile and pre-determined ground truth, which may serve as the ultimate standard. To assess accuracy, specifically for MRV, the presence or absence of amino acid mutations above these different reporting thresholds can be detected and true/false positive/negative measures calculated as the matching or mismatching with the ground truth that is linked to the sample data. This will allow accuracy measures calculation and validation of specific software when reporting drug resistant variants at different thresholds. In addition, a linear correlation between the obtained frequencies of amino acid mutations and the ground truth mutations can also be calculated to obtain generic linear correlation measures and assess the precision of the tool being evaluated.

Finally, HIVDR reporting of the obtained results in a standardized format is critical for the ability to exchange and validate results obtained from a dry panel or real data. Systematic EQA programs will benefit from the standardized format. The First Winnipeg Consensus recommended AAVF format specification as an additional report that would be useful for automatic result validation. This format includes codon coverage, mutation frequencies, reference, and the analysis features that are needed for traceable result validation. Therefore, reference AAVF outcomes from the prospective dry panels should be provided as part of the ground truth for pipeline validation or EQA assessment purposes, along with automatic parsing and comparison tools.

## 6. Conclusions

EQA capacity building programs, as well as software development for NGS-based HIV-DRT, will benefit from strategically designed and well-characterized dry panels that remain to be established. Many challenging but essential requirements need to be taken into account while constructing such dry panels. With the increasing adoption of NGS HIV-DRT worldwide, more research and development efforts are desired for the construction, validation and appropriate application of these panels, especially to serve the needs for effective EQA for such assays.

## Figures and Tables

**Table 1:** Specific dataset and software features to be included and validated by next generation sequencing (NGS) HIV drug resistance testing (DRT) dry panels.

	Type of Data	Strategy for Inclusion
**Sequence Quality Control**		
Minimum quality score = 25 (Error probability = 0.3%)	Real Data Synthetic Data	Include sample with insufficient quality Include low quality sequence reads with SM
Minimum read length = 75 bp pair	Synthetic Data	Include short good quality reads with SM
Contamination control	Synthetic Data Real Data	Include non-viral contamination Include cross-contaminated pair
APOBEC mutation check	Forged Data	Include APOBEC signature codons or hypermutated reads with SM
**Sequence Alignment Strategy**		
Allow use of HXB2 reference		Check for content
Whole *pol* gene sequencing	Real Data	Include PR, RT, and IN genes data, check for content
Management of InDels	Synthetic Data	Include samples with in-frame full-codon InDels
Codon-aware alignment	Synthetic Data	Include samples with miss-aligning InDels
**Variant Calling Quality Control**		
Codon level variant calling	Synthetic Data	Include within-codon nucleotide mixes to discard nucleotide-level variant calling
Variant count	Synthetic Data	Include controlled number of reads with specific mutation
Variant depth of coverage	Real Data	Design specific region with limited depth of coverage
**HIVDR Reporting**		
Software/pipeline version		Check for content
Consensus sequence export 15%	Synthetic Data	Include mixes >20%
Quantitative AAVF export		Check for content format compliance
**Accuracy/Precision Assessment**		
Consensus sequence similarity	Real Data	Calculate phylogenetic distance vs. ground truth, establish threshold
Mutation correlation	Synthetic/Forged Data	
TP/TN/FP/FN	Synthetic/Forged Data	Calculate accuracy at different thresholds vs. ground truth

SM, Specific (target) mutations that can be used for flagging; PR, Protease; RT, reverse transcriptase; IN, integrases; TP, true positive; TN, true negative; FP, false positive; FN, false negative.
